# The Role of Total Parenteral Nutrition in Patients with Peritoneal Carcinomatosis: A Systematic Review and Meta-Analysis

**DOI:** 10.3390/cancers13164156

**Published:** 2021-08-18

**Authors:** Xing-Yi Sarah Ong, Rehena Sultana, Joey Wee-Shan Tan, Qiu Xuan Tan, Jolene Si Min Wong, Claramae Shulyn Chia, Chin-Ann Johnny Ong

**Affiliations:** 1 Department of Sarcoma, Peritoneal and Rare Tumours (SPRinT), Division of Surgery and Surgical Oncology, National Cancer Centre Singapore, Singapore 169610, Singapore; sarah@u.duke.nus.edu (X.-Y.S.O.); joey.tan.w.s@nccs.com.sg (J.W.-S.T.); qiu.xuan@nccs.com.sg (Q.X.T.); jolene.wong.s.m@singhealth.com.sg (J.S.M.W.); claramae.chia.s.l@singhealth.com.sg (C.S.C.); 2 Department of Sarcoma, Peritoneal and Rare Tumours (SPRinT), Division of Surgery and Surgical Oncology, Singapore General Hospital, Singapore 169608, Singapore; 3 Duke-NUS Medical School, Singapore 169857, Singapore; rehena.sultana@duke-nus.edu.sg; 4 Laboratory of Applied Human Genetics, Division of Medical Sciences, National Cancer Centre Singapore, Singapore 169610, Singapore; 5 SingHealth Duke-NUS Oncology Academic Clinical Program, Duke-NUS Medical School, Singapore 169857, Singapore; 6 Institute of Molecular and Cell Biology, A*STAR Research Entities, Singapore 138673, Singapore

**Keywords:** total parenteral nutrition, peritoneal carcinomatosis, peritoneal metastases, peritoneal disease

## Abstract

**Simple Summary:**

Patients with peritoneal carcinomatosis often develop complications which prevent them from receiving adequate oral intake. This can contribute to malnutrition, as well as increased morbidity and mortality. In such patients, total parenteral nutrition, which involves the intravenous administration of nutrients thereby bypassing the oral route, can be lifesaving. In this study, we performed a systematic review and meta-analysis of the existing literature to evaluate the effect of total parenteral nutrition on the survival of patients with peritoneal carcinomatosis. In light of the limited treatment options available, total parenteral nutrition may improve survival outcomes, but further studies are needed to conclude definitively.

**Abstract:**

Peritoneal carcinomatosis (PC) is often associated with malnutrition and an inability to tolerate enteral feeding. Although total parenteral nutrition (TPN) can be lifesaving for patients with no other means of nutritional support, its use in the management of PC patients remains controversial. Therefore, a systematic review and meta-analysis was performed to evaluate the benefit of TPN on the overall survival of PC patients, in accordance with PRISMA guidelines. A total of 187 articles were screened; 10 were included in this review and eight were included in the meta-analysis. The pooled median overall survival of patients who received TPN was significantly higher than patients who did not receive TPN (*p* = 0.040). When only high-quality studies were included, a significant survival advantage was observed in PC patients receiving TPN (*p* < 0.001). Subgroup analysis of patients receiving chemotherapy demonstrated a significant survival benefit (*p* = 0.008) associated with the use of TPN. In conclusion, TPN may improve survival outcomes in PC patients. However, further studies are needed to conclude more definitively on the effect of TPN.

## 1. Introduction

Peritoneal carcinomatosis (PC) refers to the metastatic involvement of the peritoneum, typically secondary to gastrointestinal, gynecological, or other rare malignancies. It is often associated with poor prognosis, disease progression [1], and high rates of malnutrition [2], which are in turn associated with increased morbidity and mortality [3]. It is particularly difficult to treat as it is known to respond poorly to systemic chemotherapy [4,5], even compared to metastatic disease to other sites [6]. Palliative attempts to debulk tumor burden are challenging [5] and rarely confer an increase in overall survival [7], especially in nongynecological cancers; key factors deterring resection include tumor burden, response to chemotherapy, and length of the disease-free interval. With regard to gynecological cancers, the results of recent studies suggest that secondary cytoreductive surgery (CRS) may be beneficial to survival in highly selected patients with recurrent ovarian cancer if complete cytoreduction can be achieved [8], but this benefit has not yet been demonstrated in unselected patient populations [9] or in patients with incomplete resection [8]. PC was regarded as a terminal condition [5] until the advent of cytoreductive surgery and hyperthermic intraperitoneal chemotherapy (CRS-HIPEC), a potentially curative option currently being evaluated in clinical trial [10,11] along with other recent developments such as pressurized intraperitoneal aerosol chemotherapy (PIPAC), which has produced promising results [12]. However, only a highly selected group of patients are candidates for CRS-HIPEC, which requires extensive and complex bowel work. Malnutrition also affects patients’ eligibility and tolerance for CRS-HIPEC [13], as its effect on the immune function often leads to an increase in postoperative infection rates, complications in wound healing, and length of hospital stays [14].

Total parenteral nutrition (TPN) involves intravenous administration of complex nutritional formulae in the absence of other significant intake of nutrition. Its use in clinical practice remains controversial as it is costly and labor-intensive, and it has been associated with serious infectious and metabolic complications [15,16,17]. In patients who are able to tolerate an oral diet, it has been shown to cause increased complications with no benefit in oncological outcomes [18]. However, it can be lifesaving in patients with no other means of nutritional support [19]; current guidelines recommend its use in surgical and nonsurgical cancer patients who are malnourished or likely to be unable to eat for more than 7 days [18,20].

Patients with PC may be candidates for TPN as malnutrition often results not only from metabolic effects of the tumor burden [21], but also from difficulties in enteral feeding caused by complications secondary to peritoneal involvement such as malignant bowel obstruction (MBO) [22] and ascites [23]. Associated symptoms such as abdominal pain, nausea, and vomiting are also exacerbated by the intake of food [13,22]. Furthermore, compared to other gastrointestinal surgical procedures, CRS-HIPEC has been associated with longer postoperative ileus and inability to eat [24]. However, the indications, benefit, and utility of TPN in patients with PC remain unclear, and evidence for the effectiveness of TPN is generally considered to be lacking [18,25].

Although multiple reviews discussed the role of TPN in patients with inoperable MBO [25,26], advanced cancer patients [27,28], and critically ill patients [29], these studies investigated the utility of TPN in prolonging survival in more varied patient populations, including end-stage patients with various forms of advanced cancer, whose indications for TPN include cancer cachexia in addition to bowel obstruction and, in one study, trauma and sepsis patients, who may still be able to take in oral nutrition [29]. Currently, none address the role of TPN in the distinct challenges faced by PC patients specifically. Due to the particular importance of extensive surgical intervention and the prevalence of complications that could interfere with enteral feeding, TPN could be invaluable in improving their status at various phases of treatment, namely, curative resection, palliative treatment, and supportive care. This study aimed to evaluate the benefit of TPN versus no nutritional support on overall survival (OS) in patients with PC in a systematic review and meta-analysis of the existing literature. Secondary aims included comparing the rate of complications in patients receiving TPN versus no TPN and exploring the effect of TPN on patients’ quality of life (QOL).

## 2. Materials and Methods

This study was conducted in accordance with Preferred Reporting Items for Systematic Reviews and Meta-Analyses (PRISMA) guidelines [30] and registered with ResearchRegistry (UIN: reviewregistry1169). Relevant primary studies were systematically searched for using keywords and database-specific index terms for “total parenteral nutrition”, “peritoneal carcinomatosis”, “peritoneal metastases”, and “peritoneal disease” in PubMed, MEDLINE (Ovid), Embase, CINAHL, Web of Science, and Scopus (up to 9 October 2020), with filters applied for human subjects and English-language papers. References from identified studies and relevant reviews were also screened.

Inclusion criteria for articles were as follows: (1) patients diagnosed with PC regardless of primary tumor type, (2) TPN administration as part of the intervention, and (3) reporting of OS as an outcome of the study. Exclusion criteria were as follows: (1) TPN used only as a measure of treatment outcome, (2) relationship between TPN and outcome data could not be determined, (3) articles not written in English, and (4) reviews, editorials, conference proceedings, case reports, case series with <5 patients [31,32], and animal studies.

Titles and abstracts were screened according to predetermined selection criteria, and the remaining articles were subjected to full-text screening.

The following information was extracted: basic information (authors, title, publication year, country, type of study), participant characteristics (sample size, period of treatment, age, gender, performance indicators, nutrition status), disease factors (site of primary tumor, tumor histology, stage of cancer), interventions (TPN protocol, other cointerventions, prior treatment received), and outcomes (OS, complications and QOL as quantified in questionnaires such as the EORTC Core Quality of Life questionnaire (EORTC QLQ-C30)) [33]. Corresponding authors were contacted when further data were required for analysis.

Risk of bias was assessed as low, moderate, or high using the Newcastle–Ottawa scale (NOS) [34] for nonrandomized studies based on cohort selection, comparability, and outcomes. The revised Cochrane Risk of Bias tool (RoB 2) [35] was used to assess randomized studies in the domains of randomization, deviation from the intended intervention, incomplete outcome data, measurement of outcomes, and selective reporting. Selection, screening, data extraction, and risk of bias assessment was done by two independent reviewers (X.-Y.S.O. and J.W.-S.T.); if no consensus was reached, the final decision was made by a third independent reviewer (Q.X.T.).

Meta-analysis was performed for all included studies with sufficient available data. Meta-analyses of pooled median OS, complications, and QOL were specified to be performed for patients with TPN versus no TPN, with further subgroup analyses to be done based on primary tumor type and treatment received if at least two studies were identified in two subgroups.

Published summary statistics were used for meta-analysis due to the lack of individual patient data (IPD). As the meta-analysis included single-arm studies, median values were pooled using the inverse variance method, similar to approaches used by Lueza et al. [36] and Wei et al. [37]. The random effects model was chosen due to expected heterogeneity between studies; 95% confidence intervals (95% CI) and *p*-values were also calculated.

Heterogeneity was assessed using the I^2^ statistic and χ^2^ test. If evidence of substantial heterogeneity was found, sensitivity analysis excluding studies with higher risk of bias would be performed. Publication bias was assessed with a funnel plot. Any *p*-values less than 0.05 were considered significant, and analysis was performed using Review Manager 5.4 (RevMan) [38].

## 3. Results

### 3.1. Study Selection and Participant Characteristics

**Figure 1:**
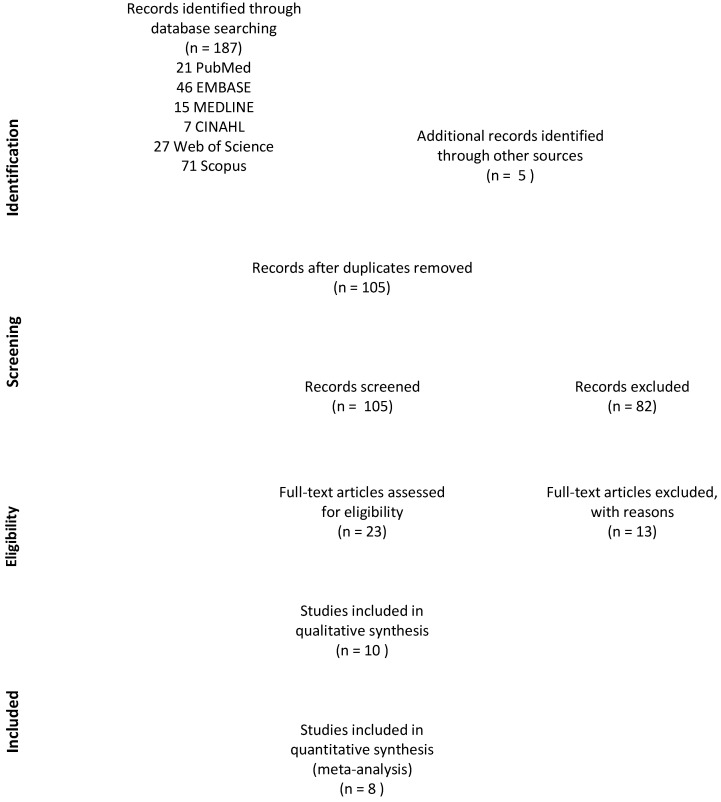
PRISMA flow chart [30].

A total of 192 articles were identified in a search of six databases, including five articles identified from references of relevant papers (Figure 1). After removing 87 duplicates, 105 articles were screened and 23 were shortlisted for full-text review. A total of 10 articles were included in the qualitative synthesis; eight included sufficient statistical data for meta-analysis.

Seven studies were retrospective, with five cohort studies [15,39,40,41,42] and two case series [43,44]. The three prospective studies included a randomized controlled trial (RCT) [45], cohort study [46], and case series [47] (Table 1). All participants were diagnosed with PC except for 51 with gastrointestinal malignancy included as a control for the effects of PC [42]; data were not extracted for this group of patients. These studies comprised a total of 1660 participants, with 620 included in the meta-analysis. 

Only one study involved patients who received TPN after CRS-HIPEC [46]. The remaining nine comprised patients with unresectable MBO and were divided among studies in which all patients received chemotherapy [39,43], none received chemotherapy [42,44,45], and a mix of both [15,40,41,47]. There was insufficient information to divide the third group into patients receiving versus not receiving chemotherapy.

Characteristics of study participants are summarized in Table 2. Overall, 51.7% were female and 43.7% were male, with no available data for 4.5%. The age range was wide; median values ranged from 55 to 60 (range 17–88) years, and means ranged from 52.3 (range 33–65) to 60 (SD 13) years.

Primary tumor sites could be grouped into gastrointestinal (1235), gynecological (386), and others (39). Three studies provided further information about tumor histology [39,43,46]. Three provided a summary of the staging of participants [39,40,41], while another three used less specific terms such as “advanced” or “terminal” [42,44,45].

Detailed information on TPN regimens was provided in two studies [45,47]. Two stated that nutritional support was personalized according to individual patient requirements [42,44], while six did not provide details of TPN composition [15,39,40,41,43,46]. Duration of TPN administration was reported in four studies and, in two, ranged from a mean of 24.1 (SD 27.4) to 60.70 (SD 44.49) days [42,47]; the other two reported a median of 45 (range 9–639) days [43] and an average of 10 days postoperatively [46].

Cointerventions were reported in seven studies, including concurrent chemotherapy in six [15,39,40,41,43,47], gastrostomy tube placement in four [15,39,40,41], and CRS-HIPEC in one [46].

A total of 13 studies were excluded from full-text screening (Table 3). Six [48,49,50,51,52,53] included TPN dependency only as a measure of complications or assessment of outcomes, while, in seven [24,54,55,56,57,58,59], the relationship between TPN and outcomes data could not be determined.

With regard to risk of bias, four nonrandomized studies were determined to be of high quality, and the other five were determined to be of moderate quality (Table 4). There were some concerns regarding the risk of bias for the single RCT, largely due to the lack of information about the randomization process (Table 5).

As fewer than 10 studies were included in the meta-analysis, funnel plot analysis was not done as per Cochrane recommendations [60].

### 3.2. Primary Outcome Measures: Overall Survival

All studies reported OS outcomes. However, definitions for survival differed, including time from gastrostomy tube placement to death in three [15,39,40], time from start of TPN to death in two [43,47], and time from diagnosis of terminal MBO to death in one [41]. Four did not provide a definition of OS [42,44,45,46].

**Figure 2:**
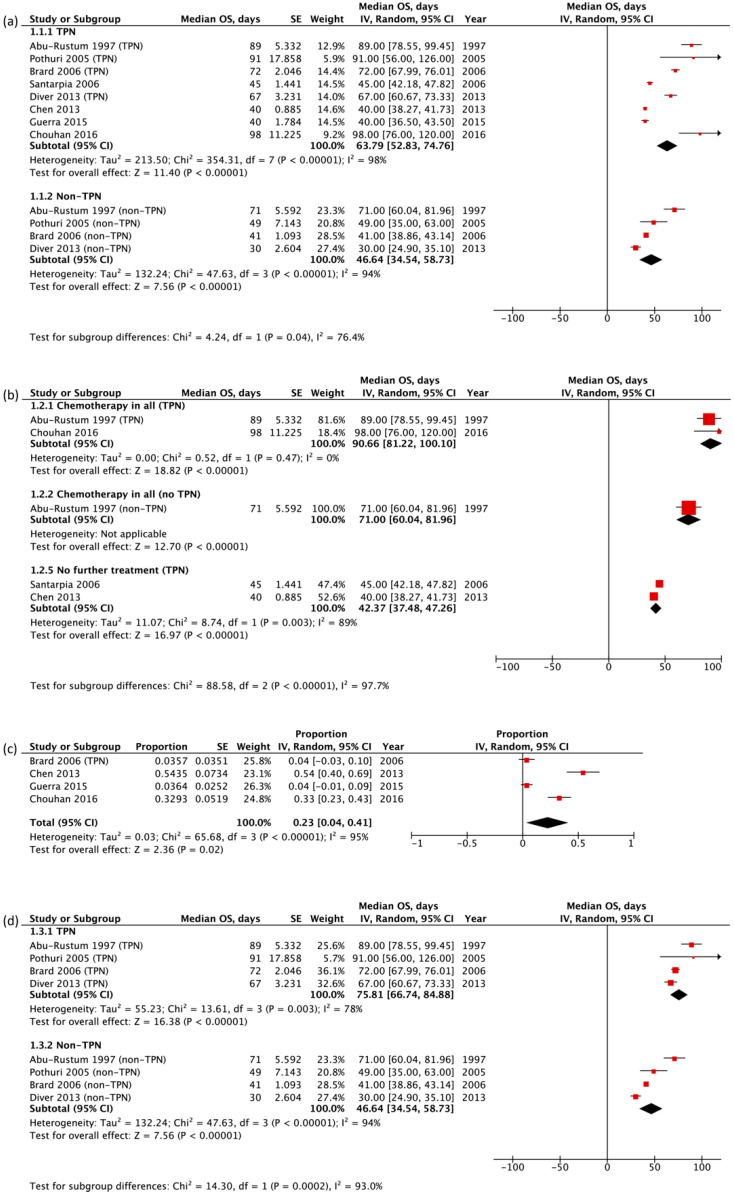
(**a**) Forest plot showing subgroup analysis of the median survival time based on TPN status, i.e., TPN and non-TPN. Pooled subgroup median survival time was compared between TPN and non-TPN groups. (**b**) Forest plot showing subgroup analysis of the median survival time based on TPN status, i.e., TPN and non-TPN among patients receiving chemotherapy. Pooled subgroup median survival time was compared between TPN and non-TPN groups. (**c**) Forest plot showing pooled complication rate with 95% CI. (**d**) Forest plot showing subgroup analysis of the median survival time based on TPN status, i.e., TPN and non-TPN in high-quality studies. Pooled subgroup median survival time was compared between TPN and non-TPN groups. In (**a**,**b**,**d**) forest plots, squares indicate the individual median survival time. In (**c**), squares indicate the individual complication rate. In all figures, bars represent 95% CIs of each included study. The size of each square is proportional to the percentage weight of the individual study in the meta-analysis. Black diamonds indicate the pooled median survival time in (**a**,**b**,**d**) or pooled complication rate in (**c**) and 95% CI.

Eight studies reported median OS outcomes and were included in the meta-analysis [15,39,40,41,42,43,44,47]; all eight involved PC patients with inoperable MBO. The pooled median OS of patients receiving TPN and not receiving TPN was 63.79 (95% CI: 52.83–74.76) days and 46.64 (95% CI: 34.54–58.73) days, respectively, with a significant survival benefit of 17.15 days (*p* = 0.040) (Figure 2a). Significant statistical heterogeneity was observed (χ^2^ = 424.74, df = 11, *p* < 0.001, I^2^ = 97%), and subgroup analyses were conducted to explore possible sources of heterogeneity.

Subgroup analysis based on treatment included only studies in which either all [39,43] or no patients [42,44] received chemotherapy (Figure 2b). For studies in which all participants received chemotherapy, the pooled median OS for the group receiving TPN and not receiving TPN was 90.66 (95% CI: 81.22–100.10) days and 71.00 (95% CI: 60.04–81.96) days, respectively, with a significant difference of 19.66 days (*p* = 0.008). For studies in which no patients received chemotherapy, only data for the group receiving TPN were available for meta-analysis; the pooled median OS was 42.37 (95% CI: 37.48–47.26) days. For patients receiving TPN, chemotherapy conferred a significant survival benefit of 48.29 days (*p* < 0.001). However, even in the subgroup analysis, the I^2^ remained high.

### 3.3. Additional Outcome Measures: Complications, Quality of Life (QOL)

Eight studies reported complications in participants. Comparing all complications in patients with TPN versus no TPN as originally planned was not possible as none of the double-arm studies distinguished complications in these groups. As cointerventions were potential confounders, quantitative analysis was only done for complications specifically attributed to TPN by study authors [41,42,43,47]. The pooled proportion of participants who experienced complications specifically attributed to TPN was 23% (95% CI: 4–41%) (Figure 2c). Of these four studies, only one reported a median follow-up time of 89.5 (range 4–2117) days [43].

There was insufficient information to conduct statistical analysis on QOL measures, as only one study investigated QOL as measured by Karnofsky Performance Status (KPS) before and after TPN [44]; other studies included only a qualitative discussion of QOL.

### 3.4. Sensitivity Analysis

Sensitivity analysis was performed by repeating analyses on the four high-quality studies identified using NOS [15,39,40,41]. As there were no significant differences in the primary outcome measures, the results were considered to be robust (Figure 2d).

Sensitivity analysis could not be done for treatment-based subgroup analyses as only one study had all patients receiving chemotherapy, while the other three had a mix of patients receiving and not receiving chemotherapy. Similarly, only one study reported complications specific to TPN.

## 4. Discussion

Clinically, patients on oncological therapy are considered distinct from patients no longer receiving treatment; TPN may improve or maintain their general condition, permitting them to receive further therapy [22], or allow waiting for gut function to be restored [25]. In the latter, TPN may prolong their lifespan by mitigating the effects of malnutrition [22]. Thus, three groups of patients who might benefit from TPN were identified: (1) patients undergoing extensive bowel work (CRS-HIPEC), (2) patients with MBO undergoing chemotherapy, and (3) patients on supportive care no longer receiving oncological therapy.

Our primary objective was to evaluate the effect of TPN versus no nutritional support on OS in patients with PC. A meta-analysis of eight eligible studies demonstrated a significant survival benefit of 17.15 days (*p* = 0.040) in patients with MBO who received TPN versus no TPN. When this analysis was repeated with high quality studies only, this difference persisted with a significant survival benefit of 29.17 days (*p* < 0.001) in patients who received TPN versus no TPN. This significant survival benefit is in agreement with existing studies citing an apparent survival benefit associated with TPN [61]. Due to variations in primary disease sites, concurrent interventions, and patient factors, further subgroup analyses were performed to explore these differences.

Tumor biology is thought to play an important role in the prognosis of patients with PC. Compared to PC of non-gynecological origin, PC from gynecological primaries, particularly ovarian cancer, is thought to be more amenable to surgical intervention [13] and chemotherapy, with a better immediate prognosis [62,63]; this advantage decreases with disease recurrence and multiple lines of chemotherapy [64,65]. In contrast, PC of gastrointestinal origin is associated with a poor prognosis [66,67,68], although recent advances in more aggressive treatments such as CRS-HIPEC have resulted in improved survival [67]. In the studies included in this review, colorectal, gastric, and pancreatic cancers [23,66,68] were the more common subtypes associated with PC. One study included patients with pseudomyxoma peritonei (PMP) from appendiceal mucinous tumors, who have much better long-term outcomes after CRS-HIPEC than patients with other gastrointestinal primaries [67,69]. However, due to the lack of information on outcomes corresponding to specific primary tumor sites within individual studies, subgroup analysis based on primary tumor sites could not be performed.

We also aimed to evaluate if patients who received chemotherapy had better survival outcomes, as a surrogate measure of treatment intent in the palliative setting. All studies comprising a mix of patients receiving and not receiving chemotherapy found that chemotherapy conferred a survival benefit [15,41,47], although, in one study, this was restricted to patients younger than 55 with no liver metastases [40]. Similarly, our study demonstrated that chemotherapy conferred a statistically significant survival benefit of 48.29 days (*p* < 0.001) in patients receiving TPN (Figure 2b). Comparing patients who received chemotherapy and TPN versus chemotherapy alone, TPN was associated with a significant survival benefit of 19.66 days (*p* = 0.008). The pooled median OS for studies with participants receiving TPN only was 42.37 (95% CI: 37.48–47.26) days, with no data available for patients who received neither TPN nor chemotherapy. However, Solassol et al.’s RCT [45] demonstrated that TPN conferred a significant survival benefit of 39 days (mean OS) in patients who received only symptomatic treatment. Taken together, these results appear to support the finding that TPN has a positive effect on the survival of PC patients, independent of the effect of chemotherapy.

Factors other than survival outcomes may be critical in evaluating the utility of long-term TPN in patients unlikely to regain the ability to eat. QOL has increasingly become a major focus of care in cancer patients [70,71]; proponents believe that TPN is justifiable only if it can improve patients’ QOL beyond merely prolonging their lives [16,47,72], as associated complications [18] and time spent on TPN infusion may detract from patients’ desired use of time. Our secondary aim was, therefore, to evaluate the effect of TPN on complications and QOL.

TPN has been associated with potentially life-threatening complications such as catheter-related infections, thrombosis, liver-related complications, and metabolic imbalances [73]. In our study, catheter-related bloodstream infections, reported in 45 of 211 patients, were the most common TPN-related complication, with severe infection necessitating termination of TPN in five. Hyperbilirubinemia was reported in 10 patients. However, insufficient information on follow-up time made it difficult to compare the findings to complication rates reported in existing literature. Although TPN may have played a role in other complications, comparisons could not be made as none of the double-arm studies distinguished complications in the TPN group from those in the non-TPN group.

No studies evaluated patients’ QOL before and after receiving TPN using QOL-specific questionnaires. Santarpia et al. [44] reported that, in a majority of patients, functional (KPS) and nutritional parameters stabilized or improved after a month of home PN, suggesting a positive effect on QOL. In contrast, Chouhan et al. [43] observed that high morbidity rates and time spent in the hospital were likely to negatively impact QOL. No conclusions could be drawn from two studies reporting ECOG status prior to but not post TPN [41,47].

Limited data were available for analysis in patients who underwent extensive bowel resection. The single study in this category reported outcomes of patients with PMP who received surgical resection and standard postoperative TPN, including OS and Clavien–Dindo complications [46]. However, as neither a control group for TPN nor any other studies in this category were available for comparison, no conclusions on the effect of TPN could be made. Due to the lack of median OS data, the different clinical contexts, and patient prognosis, it was not meaningful to pool results with the other studies. Even so, there may be a role for TPN in the management of these patients. Investigating the effect of differing perioperative care procedures on CRS-HIPEC patients, Elekonawo et al. [24] reported that, due to slow gastrointestinal recovery, postoperative TPN was often unavoidable, even in centers where early enteral feeding was favored. Similarly, Vashi et al. [54] demonstrated that preoperative nutrition status was associated with length of stay and OS, a finding supported by the existing literature [74]. In the absence of standardized evidence-based perioperative care procedures for CRS-HIPEC [75], further investigations would be useful in characterizing the benefit of TPN in these patients.

Qualitative analysis yielded two potential areas for investigation. Firstly, platinum analogs combined with a taxane, the current standard of care for ovarian cancer [62,65], have been shown to be less effective in patients who have received multiple lines of chemotherapy due to the development of platinum resistance [65]. Data from two studies [15,39] suggests that, for PC patients with primary ovarian cancer, chemotherapy-naïve patients with MBO on first presentation may be more likely to benefit from TPN and chemotherapy. Secondly, the effect of TPN on QOL and nutritional status is believed to be correlated with the time period of receiving TPN, i.e., the longer a patient receives TPN, the more likely they would experience improved QOL and nutritional status [45,73]. Given the poor prognosis of PC patients, prospectively identifying patients likely to have a longer OS who stand to gain a greater benefit from TPN remains a challenge [16,76,77]. Two studies suggest that BMI [42], pain, KPS, albumin, and cholinesterase levels [44] may predict survival. These findings may warrant further study.

## 5. Conclusions

In conclusion, the benefit of TPN remains closely determined by the tumor biology and baseline health status of PC patients. This systematic review and meta-analysis found a small but significant difference in survival between PC patients with MBO given TPN versus no TPN. Further subgroup and qualitative analyses suggested that this benefit persisted regardless of chemotherapy administration. However, differences in disease characteristics, patient factors, and even definitions of OS which may have affected this outcome could not be further explored due to the lack of IPD, and heterogeneity remained high in the treatment subgroup analysis. In particular, the lack of level 1 evidence from RCTs made the results highly susceptible to selection bias, with initiation of TPN largely based on physician or patient preference. However, given the complexity of decision making in PC patients, conducting randomized studies will likely be difficult, and TPN will most probably continue to be given on the basis of joint decisions by patients and physicians.

It is our view that TPN should continue to be offered as an option in patients otherwise unable to take an oral diet, provided institutions possess the necessary resources and expertise to implement it. In addition, attention to patients’ preferences and QOL is critical as, once initiated, patients and caregivers may find it difficult to withhold TPN despite increasing clinical deterioration and patient discomfort [16,47,72] due to fear of death [16,78]. Given the limited prognosis of the study group, the survival benefit conferred by TPN, although modest, may be crucial for some patients and, in certain cases, well worth the potential cost. Nevertheless, continued investigations, especially in the area of complications and QOL measures, are needed to allow for better clarity in decision making. In particular, the use of TPN in CRS-HIPEC patients is clearly an underdeveloped field of study, and much more needs to be done to evaluate its role.

## Figures and Tables

**Table 1:** Characteristics of all included studies (*n* = 10).

Author	Country	Study Design	Sample Size (*n*)	TPN (*n*)	Median OS (Days)	Cointerventions (*n*)	Complications (*n*)
Abu-Rustum et al., 1997 [39]	USA	Retrospective cohort study	21	Yes: 52% (11)	89	Drainage gastrostomy tube 100% (21) Chemotherapy 100% (21) - Paclitaxel (8) - Platinum-based (7) - Other third-line chemotherapy (6) Second salvage iv chemotherapy regimen (3)	Gastrostomy-related complications in 33% (7) Replacement of new drainage tube required in 24% (5) Chemotherapy-related complications, nadir fever or sepsis requiring readmission in 24% (5)
				No: 48% (10)	71		
Pothuri et al., 2005 [40]	USA	Retrospective cohort study	94	Yes: 15% (14)	91	PEG tube 100% (94) Chemotherapy 31% (29)	PEG tube placement-related complications in 18% (17)
				No: 85% (80)	49		
Brard et al., 2006 [41]	USA	Retrospective cohort study	55	Yes: 51% (28)	72	Concurrent chemotherapy in patients receiving TPN Yes: 64% (18) No: 36% (10)	Line sepsis in 4% (1) Gastrostomy tube replacement required in 12.5% (2)
				No: 49% (27)	42	Concurrent chemotherapy in patients not receiving TPN Yes: 24% (7) No: 76% (20)	
Diver et al., 2013 [15]	USA	Retrospective cohort study	115	Yes: 36% (41)	67	Concurrent chemotherapy in patients receiving TPN Yes: 54% (22) No: 46% (19)	Gastrostomy-related complications in 45% (51)
				No: 63% (74)	30	Concurrent chemotherapy in patients not receiving TPN Yes: 31% (23) No: 69% (51)	
Guerra et al., 2015 [47]	Spain	Prospective case series	55	Yes: 100% (55)	40	Able to further receive chemotherapy after TPN Yes: 51% (28) No: 49% (27)	Catheter-related bloodstream infections in 3.6% (2) No thrombotic episodes/severe metabolic complications
Chouhan et al., 2016 [43]	USA	Retrospective case series	82	Yes: 100% (82)	93	Chemotherapy 100% (82)	Line infections in 20.7% (17) Hyperbilirubinemia in 12.2% (10) Bowel perforation in 4.9% (4)
Solassol et al., 1979 [45]	France	Randomized controlled trial	40	Yes: 53% (21)	46 (mean)	(Steroids (symptomatic) only)	-
				No: 47% (19)	7 (mean)		
Santarpia et al., 2006 [44]	Italy	Retrospective case series	152	Yes: 100% (152)	45	Analgesics 44.1% (67) Antiemetics 27% (41) Nasogastric tube 8.6% (13)	-
Chen et al., 2013 [42]	Taiwan	Retrospective cohort study *	46	Yes: 100% (46)	40	-	Sepsis related to TPN in 54.3% (25) - Severe infection leading to termination of TPN in 10.8% (5)
Ansari et al., 2016 [46]	UK	Prospective cohort study *	980	Yes: 100% (980)	CCRS: 3102 MTD: 1596	CCRS + HIPEC 75.3% (738) MTD ± HIPEC 24.7% (242)	Clavien–Dindo grade 30 days post-op CCRS: - I and II, minor morbidity in 74.2% (548) - III and IV, major morbidity in 15.2% (112) - V, mortality in 0.8% (6) MTD: - I and II, minor morbidity in 84.2% (204) - III and IV, major morbidity in 14.5% (35) - V, mortality in 1.7% (4)

* Control arms were not relevant to the primary outcome of this study. Abbreviations: TPN, total parenteral nutrition; PEG, percutaneous endoscopic gastrostomy; CCRS, complete cytoreductive surgery; MTD, maximal tumor debulking; HIPEC, hyperthermic intraperitoneal chemotherapy.

**Table 2:** Characteristics of all patients included in qualitative analysis (*n* = 620).

Author	Period of Treatment	Median Age, Years	Gender, % Female	Disease Characteristics Site of Primary Tumor (*n*) Tumor Histology (*n*) Stage of Cancer (*n*)	Prior Treatment Received (*n*)	Performance Indicators	Nutrition Status
Abu-Rustum et al., 1997 [39]	1990–1995	54.5 (mean)	100	*Site* Gynecological: epithelial ovarian (21) *Histology* Poorly differentiated adenocarcinoma (14) Moderately differentiated tumor (7) *Stage* Stage IIB (1) Stage IIIC (16) Stage IV (3) Not surgically staged (1)	Chemotherapy (18) - Median no. of regimes (range): 3 (2–6) No prior treatment (3)	-	-
Pothuri et al., 2005 [40]	1995–2002	56 (mean)	100	*Site* Gynecological: ovarian (94) *Stage* Stage I (1) Stage II (2) Stage III (66) Stage IV (25)	Previous lines of chemotherapy - 0–2: 11% (10) - 3: 12% (11) - 4: 15% (14) - 5: 15% (14) - 6: 13% (12) - 7: 5% (5) - 8: 12% (11) - 9+: 18% (17) No. of prior laparotomies - 1: 36% (34) - 2: 41% (39) - 3+: 22% (21) Initial debulking - Optimal 48% (45) - Suboptimal 51% (48)	-	-
Brard et al., 2006 [41]	1994–2002	56.4 (mean)	100	*Site* Gynecological: epithelial ovarian (55) *Stage* Stage IIIC/ IV (55)	CRS at time of original diagnosis (55) Platinum-based chemotherapy (paclitaxel/ platinum) (55)	ECOG (*n*) TPN: 1 (1) 2 (23) 3 (14) No TPN: 1 (0) 2 (24) 3 (3)	Albumin (g/dL), mean (SD) All: 2.47 (0.72) TPN: 2.52 (0.74) No TPN: 2.41 (0.71)
Diver et al., 2013 [15]	2000–2008	57	100	*Site* Gynecological - Ovarian/fallopian tube/peritoneal (96) - Cervical (6) - Uterine (13)	No. of lines of chemotherapy (115) - 1: 17% (20) - 2: 19% (22) - 3+: 58% (67) - Unknown: 5% (6)	-	-
Guerra et al., 2015 [47]	2007–2012	60 (mean)	-	*Site* Gastrointestinal (28) Gynecological (10) Others (7)	Previous lines of chemotherapy, mean (SD) - GI: 1.82 (±1.04) - Gy: 2.20 (±1.14) - Others: 1.43 (±0.53)	Baseline ECOG, mean (SD): 1.5 (0.5)	BMI (kg/m^2^), mean (SD): 21.6 (±4.3) Malnutrition in 85% using MUST
Chouhan et al., 2016 [43]	2005–2013	55	62.2	*Site* Gastrointestinal (49) - Colorectal (20) - Appendix (6) - Pancreas (6) - Others (11) Gynecological (18) - Ovarian/Primary peritoneal (16) - Uterine (2) Others (15) *Histology* Carcinoma (71) Non-carcinoma (11)	Abdominal surgery (59) Previous lines of chemotherapy - 0: (38) - 1: (15) - 2+: (29)	-	BMI (kg/m^2^), median (range): 23.9 (14.3–38.0), *n* = 81 Albumin (g/dL), median (range): 2.8 (1.6–4.4), *n* = 79
Solassol et al., 1979 [45]	1976–1977	52.3 (mean)	62.5	*Site* Gastrointestinal (23) Gynecological: ovarian (17) *Stage* “advanced malignant disease”; no lung/liver metastases	-	-	-
Santarpia et al., 2006 [44]	1996–2003	57.8 (mean)	70.4	*Site* Gastrointestinal (90) - Gastric (48) - Colorectal (30) - Ileum (5) - Gallbladder (4) - Pancreas (3) Gynecological (49) - Ovarian (42) - Endometrial (7) Others (13) *Stage* “advanced”	-	KPS ≤ 40	Weight (kg), mean (SD), median: 53.4 (±10.9), 50.2 BMI (kg/m^2^), mean (SD), median: 20.1 (±3.6), 19.6 Albumin (g/dL), mean (SD), median: 3.1 (±0.6), 3.1
Chen et al., 2013 [42]	2013	56.5 (mean)	47.8	*Site* Gastrointestinal (35) - Gastric (18) - Colorectal (15) - Pancreas (1) - Small bowel (1) Gynecological: ovarian (7) Others (4) *Stage* “advanced/terminal”	-	-	All malnutritioned based on weight, BMI, % of standard mid-upper arm circumference and triceps skinfold thickness BMI (kg/m^2^), mean (SD): TPN: 18.6 (±3.3) No TPN: 19.5 (±3.2) Albumin (g/dL): TPN: 26 (±7.0) No TPN: 26 (±6.0)
Ansari et al., 2016 [46] (CCRS group)	2016	56	34	*Site* Gastrointestinal: appendiceal (718) *Histology* Low-grade mucinous (575) High-grade (115) Adenocarcinoma (28)	-	-	-
Ansari et al., 2016 [46] (MTD group)	2016	60	48.7	*Site* Gastrointestinal: appendiceal (231) *Histology* Low-grade mucinous (163) High-grade (50) Adenocarcinoma (18)	-	-	-

Abbreviations: CRS, cytoreductive surgery; ECOG, Easter Cooperative Oncology Group; TPN, total parenteral nutrition; BMI, body mass index; MUST, Malnutrition Universal Screening Tool; KPS, Karnofsky Performance Status; CCRS, complete cytoreductive surgery; MTD, maximal tumor debulking.

**Table 3:** Studies excluded from full-text screening.

Study	Reason(s) for Exclusion
Tsai et al., 2006 [48]	TPN dependency used as a measure of complications/outcome; intervention investigated surgery for bowel obstruction
Fajardo et al., 2012 [49]	TPN dependency used as a measure of complications/outcome, not intervention
Halkia et al., 2014 [50]	TPN dependency used as a measure of complications/outcome; intervention investigated consequences of short bowel syndrome (SBS) from CRS-HIPEC
Dineen et al., 2016 [51]	TPN dependency used as a measure of complications/outcome; intervention investigated feeding tube placement during CRS-HIPEC
Shannon et al., 2018 [52]	TPN dependency used as a measure of complications/outcome; intervention investigated gastrectomy in CRS-HIPEC
Bekhor et al., 2020 [53]	TPN dependency used as a measure of complications/outcome; intervention investigated safety of multiple reiterations of CRS-HIPEC
Vashi et al., 2013 [54]	Relationship between TPN and outcomes data could not be determined: “study not designed to investigate a causative relationship between PN and clinical outcomes”
Morris et al., 2017 [55]	Relationship between TPN and outcomes data could not be determined: TPN was investigated as a factor contributing to palliative care referral
Swain et al., 2018 [56]	No data for overall survival; complication outcomes not related to TPN
Elekonawo et al., 2019 [24]	Relationship between TPN and outcome data could not be determined: “setup of study did not allow for a fair comparison of TPN vs. early enteral feeding”
Kubi et al., 2020 [57]	Relationship between TPN and outcomes data could not be determined: TPN and surgical complications as factors of nonhome discharge
Hara et al., 2018 [58]	Relationship between TPN and outcomes data could not be determined
Osumi et al., 2018 [59]	Relationship between TPN and outcomes data could not be determined

**Table 4:** Quality assessment of nonrandomized studies using the modified Newcastle-Ottawa scale (NOS).

Study	Selection	Comparability	Outcome	Total	Quality
Abu-Rustum et al., 1997 [39]	***	*	***	*******	High
Pothuri et al., 2005 [40]	***	*	***	*******	High
Brard et al., 2006 [41]	***	**	***	********	High
Santarpia et al., 2006 [44]	***	n/a	***	******	Moderate
Chen et al., 2013 [42]	***	n/a	***	******	Moderate
Diver et al., 2013 [15]	***	**	***	********	High
Guerra et al., 2015 [47]	***	n/a	***	******	Moderate
Ansari et al., 2016 [46]	**	n/a	***	*****	Moderate
Chouhan et al., 2016 [43]	***	n/a	***	******	Moderate

Stars (*) are allocated as per NOS guidelines [34]. A maximum of 4 stars (Selection), 2 stars (Comparability) and 3 stars (Outcome) are allocated per category. n/a, not applicable.

**Table 5:** Quality assessment of randomized controlled trial using the revised Cochrane Risk of Bias tool.

Study	Risk of Bias Arising From	Risk-of-Bias Judgement
Solassol et al., 1979 [45]	The randomization process	Some concerns
	Deviations from the intended intervention	Some concerns
	Missing outcome data	Low
	Measurement of outcome	Low
	Selection of the reported result	Low
	Overall	Some concerns

## Data Availability

The data that support the findings of this study are available from the corresponding author upon reasonable request.
